# Social determinants of duration of last nursing home stay at the end of life in Switzerland: a retrospective cohort study

**DOI:** 10.1186/s12877-015-0111-3

**Published:** 2015-10-01

**Authors:** Damian Hedinger, Oliver Hämmig, Matthias Bopp, for the Swiss National Cohort Study Group

**Affiliations:** Epidemiology, Biostatistics and Prevention Institute, University of Zurich, Hirschengraben 84, 8001 Zurich, Switzerland

**Keywords:** Nursing home, Retirement home, Health services research, End-of-life care, Health inequalities, Switzerland

## Abstract

**Background:**

Due to demographic ageing and increasing life expectancy, a growing demand for long-term nursing home care can be expected. Stays in nursing homes appear to be more socially determined than hospital stays. We therefore looked at the impact of socio-demographic and health care variables on the length of the last nursing home stay.

**Methods:**

Nationwide individual data from nursing homes and hospitals in Switzerland were linked with census and mortality records. Gender-specific negative binomial regression models were used to analyze *N* = 35,739 individuals with an admission age of at least 65 years and deceased in 2007 or 2008 in a nursing home.

**Results:**

Preceding death, men spent on average 790 days and women 1250 days in the respective nursing home. Adjusted for preceding hospitalizations, care level, cause of death and multimorbidity, a low educational level, living alone or being tenant as well as a low care level at the admission time increased the risk for longer terminal stays. Conversely, a high educational level, being homeowner, being married as well as a high care level at the admission time decreased the risk for longer stays.

**Discussion:**

The length of the last nursing home stay before death was not only dependent on health-related factors alone, but also substantially depended on socio-demographic determinants such as educational level, homeownership or marital status. The support of elderly people at the admission time of a presumably following nursing home stay should be improved and better evaluated in order to reduce unnecessary and undesired long terminal nursing home stays.

**Conclusions:**

Health policy should aim at diminishing the role of situational, non-health-related factors in order to empower people to spend the last years before death according to individual needs and preferences.

## Background

Against the background of a gradually but fundamental change from acute to chronic diseases and causes of death resulting mainly from demographic ageing, a growing demand for long-term nursing and palliative care can be expected [1]. In many developed countries and ageing societies such as Switzerland or the US, staying in long-term facilities at the end of life has increased and nursing homes have been recognized as an important setting for end-of-life care [2, 3]. This trend from private home to nursing home as place of death is still ongoing and will further grow in the future [1, 2, 4]. In Switzerland, around 26 % of men and 45 % of women in 2007 and 2008 deceased in nursing homes [5], often after long stays. This is in contrast to a general preference for living and dying at home [6].

Contrary to hospitalizations for which health reasons are crucial, admissions to and stays in nursing homes may be more socially determined [7]. This is particularly true with increasing length of nursing home stay: The earlier the admission and the longer the stay in a nursing home, the more other factors than those directly related to health status may be predictive. Being male, having sufficient social (e.g., having a partner and/or adult children, particularly daughters) or material resources (e.g., owning a home) may prevent an early entry and consequently a long stay in a nursing home at the end of life [8, 9].

There is evidence for social inequalities in health and health care needs among the elderly. Numerous studies show that these inequalities persist also in old age, although they are – at least in relative terms - decreasing with age [10–15]. It is widely accepted that morbidity and mortality are dependent on socio-economic position, e.g., educational level [11, 16, 17]. However, if we look at predictors for nursing home stays, the influence of education is less clear [7, 18], but a higher household net worth appears to be associated with shorter terminal nursing home stays [19]. There is also evidence that home-owners are less likely than not-owners to be placed in nursing homes [8, 18]. Another important but hardly explored predictor of length of stays in nursing homes may be the degree of care needed and the quality of care received. In sum, there is a need for more solid evidence regarding the role and contribution of socio-economic position (SEP) for admission to and duration of stay in a nursing home.

In our explorative observational study based on linked census and administrative data, we therefore looked at potential associations between the length of the last nursing home stay and social determinants (e.g., education, homeownership, marital status, parenthood).

## Methods

### Data

We extracted data from three different sources covering basically all individuals living in Switzerland:

1. The Swiss National Cohort (SNC, www.swissnationalcohort.ch) is an anonymous linkage of census, mortality and emigration records [20]. For this study we used individual data from the 2000 census and mortality (incl. cause of death) statistics 2007–08.
2. The statistics of socio-medical institutions (SOMED) encompass nursing homes, homes for disabled, institutions for addiction patients and institutions for people with social problems. They are administered by the Swiss Federal Statistical Office and mandatory for all socio-medical institutions [21]. The institutions report limited patient information such as year of birth, sex, ZIP-code of residence, care level and date of admission and discharge. For this study we used individual data of nursing homes from the data years 2007 and 2008.
3. The medical statistics of the Swiss hospitals (MedStat), administered by the Swiss Federal Statistical Office [22]. This data source encompasses information about diagnoses, treatments, discharges and further information of hospital residents, including – for deceased persons only – full date of birth. For this study we used individual data from the data years 2002–2008. An anonymous but unique person number allows to retrieve all hospital and (since 2007) nursing home stays of the same individual.

All three data sources encompass the entire population and are fully anonymized. For reasons of data protection and confidentiality there is no common personal identification number that would allow to directly link the three data sources from hospitals (MedStat), nursing homes (SOMED), and the Swiss National Cohort (SNC) on an individual level. An anonymous common person identifier is only available for two of the three data sources (MedStat and SOMED). The linkage with the third source (SNC) had to rely on common identification variables such as place of residence, date of birth and date of death. As a consequence, among nursing home residents and hospitalized people the linkage to the SNC was only possible for those who died in an institution. The detailed linkage process is described elsewhere [5]. We restricted to individuals ≥65 years old at the admission (i.e., born before 1942) and who had died in 2007 and 2008 in a nursing home, resulting in a study population of *N* = 11,486 men and *N* = 24,253 women. Ethics approval for this study was given by the Ethics Committee of the Canton of Zurich.

### Study design

Our outcome variable was the number of days of the last nursing home stay before death. We stratified our analysis by gender because there are fundamental differences between men and women regarding institutional stays before death [8, 23–25].

**Fig. 1:**
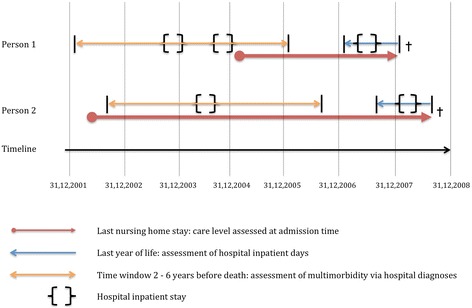
Examples of two persons with the corresponding time windows of different health variables. Data source: Swiss Federal Statistical Office, MedStat, SOMED, SNC

The independent variables were grouped into individual, familial/housing and structural/regional attributes. As control variables we included age (at the time of admission) and nationality (Swiss or foreign) as well as cause of death: malignant neoplasms (ICD 10: C00-C99), coronary heart disease (I20-I25), stroke (I60-I69), chronic obstructive pulmonary disease (COPD, J40-J47), dementia (F01, F03, G30) and all other causes combined. The care level was assessed at time of admission and can be grouped into four categories: unknown or no care needed, low (max. 40 min per day), medium (between 41 and 80 min per day) and high (more than 81 min per day). Please note that Switzerland has no homogenous care level classification for the whole country and respective information was not always complete. However, we had no alternative health variable for nursing home residents. From MedStat we derived information about multimorbidity (2+ chronic conditions), assessed from inpatient diagnoses 2–6 years before death. We defined chronic conditions using ICPC-2, of which 129 rubrics were classified as chronic conditions [26]. Additionally, we included a dummy to control for hospitalizations in the last 365 days preceding death. We used specific time windows in order to test the different impact of health indicators which are more close or more distant from death. For better understanding of our health variables, Fig. 1 presents examples of two persons with the corresponding time windows. From the 2000 census we derived the educational level according to the International Standard Classification of Education (ISCED), version 1997: no or low secondary education completed (ISCED 0–2), post-secondary non-tertiary (“medium”, ISCED 3–4), and tertiary education (“high”, ISCED 5). Also from the 2000 census we extracted information on homeownership (owner-occupier household yes vs. no) and having had children (assessed on an individual level for men and women, i.e., not necessarily the same for all couples). Marital status was assessed at the time of home admission (never married, married, widowed and divorced). Place of residence was categorized into the three main language areas of Switzerland, namely the German, French and Italian speaking parts. In order to account for geographical variation in nursing home bed availability, we included a variable with the density of nursing home beds per 100 inhabitants aged 65 years or older in 2010 on the level of 106 quite homogenous regions.

### Statistical methods

For descriptive analysis, we calculated means, frequencies and proportions of the respective variables. To point out the right-skewed distribution, we report mean, median, SD and 25th and 75th percentile of our outcome. The distribution of the outcome variable can be interpreted as a count variable. Therefore, a count regression model such as Poisson or negative binomial model is appropriate. Because of a strong overdispersion in the outcome variable, we preferred the negative binomial model over the Poisson model. Separate negative binomial regression models for both men and women, yielding estimated incident rate ratios (IRR) [27] were used to assess the impact of several independent variables on the number of days in a nursing home. For better understanding of the interpretation of IRR, we give a reading example using the estimated IRR of 1.17 for a men with “low” educational level (cf. Table 2): Compared to a man with “medium” educational level (reference category), the expected length of the last nursing home stay for a man with “low” educational level is 1.17 times higher. Negative binomial regression was used instead of Poisson regression to account for overdispersion in the data. In order to obtain global p-values for each variable in the model, we performed likelihood ratio tests. The level of significance was set to α = 0.05 (two-sided tests).

For sensitivity analysis, we also tested if some potentially relevant interaction terms (age and multimorbidity, age and cause of death, age and hospitalizations in the last 365 days preceding death) improved the model which was not the case. However, neither of those interaction terms improved the model with respect to the Bayesian information criterion (BIC). For this reason, they were omitted in the final model.

## Results

Table 1 presents the gender-specific characteristics of our study population. There are remarkable differences between the two sexes in our study population: More than 2 out of 3 individuals are women, reflecting the widely observed and often cited feminisation of ageing. Furthermore, in the average women were admitted to a nursing home in higher ages (85.2 years) than men (83.6 years). However, two out of three women in the study population are widowed compared to less than one out of three men.

**Table 1:** Characteristics of the study population

	Men		Women	
N	11,486		24,253	
Proportion (by sex)	32.1	%	67.9	%
Length of nursing home stay, mean	790.1	days	1250.2	days
Length of nursing home stay, median	383	days	849	days
Length of nursing home stay, 25th percentile	73		229	
Length of nursing home stay, 75th percentile	1098		1814	
Length of nursing home stay, SD	1072		1344	
Mean age (at time of admission)	83.6	years	85.2	years
Swiss nationality (SNC)	95.6	%	96.9	%
Cause of death (SNC):				
Cancer	18.5	%	11.5	%
Coronary hearth disease	15.7	%	15.8	%
Stroke	8.8	%	9.5	%
COPD	4.9	%	2.3	%
Dementia	15.0	%	17.8	%
Other	37.1	%	43.1	%
Care level (S):				
Low	26.8	%	28.7	%
Medium	27.1	%	28.7	%
High	41.6	%	39.4	%
Unknown/not specified	4.4	%	3.2	%
Multimorbidity (M) (assessed in time window 2–6 years before death):				
No	46.1	%	42.0	%
Yes	39.4	%	35.0	%
Unknown (no hospital stay)	14.5	%	22.9	%
Hospitalisation in the 365 days preceding death (M): yes	47.5	%	32.3	%
Educational level (SNC):				
Low	31.3	%	52.5	%
Medium	36.3	%	25.1	%
High	14.1	%	2.7	%
Unknown	18.3	%	19.6	%
House or flat owner (SNC): owner	40.9	%	30.7	%
Marital status (SNC):				
Never married	10.6	%	12.2	%
Married	53.1	%	15.8	%
Widowed	30.5	%	65.6	%
Divorced	5.9	%	6.5	%
Children (SNC):				
Yes	73.5	%	70.2	%
No	18.1	%	19.8	%
Unknown	8.4	%	10.0	%
Language region (SNC):				
German	76.4	%	73.4	%
French	19.8	%	21.8	%
Italian	3.8	%	4.7	%
Nursing home density (SNC) (beds per 100 inhabitants aged 65+)	7.1		7.0	

Data source: Swiss Federal Statistical Office, MedStat, SOMED, SNC

Sources abbreviations: S: SOMED, M: MedStat, SNC: Swiss National Cohort

The estimated incidence risk ratios (IRR) predicting the length of the last nursing home stay are presented in Table 2 for men and women separately. We added information about the mean and standard deviation according to the respective independent variable.

**Table 2:** Mean duration and results of the negative binomial regression analysis of last nursing home stay

	Men				Women			
	Length of stay (days)				Length of stay (days)			
	Mean	SD	IRR	95 % CI	Mean	SD	IRR	95 % CI
Age (at time of admission) (p < 0.001)			0.96	0.96–0.97			0.96	0.95–0.96
Nationality (p < 0.001)								
Swiss (reference)	794.6	1079.0	1.00		1252.9	1345.6	1.00	
Foreigner	692.0	893.8	0.77	0.69–0.85	1165.2	1284.3	0.83	0.76–0.89
Cause of death (p < 0.001)								
Cancer condition (ref.)	423.1	807.5	1.00		646.4	1079.4	1.00	
Coronary heart disease	835.7	1081.7	1.75	1.62–1.88	1299.2	1383.3	1.89	1.79–1.99
Stroke	926.0	1179.6	2.02	1.85–2.21	1256.4	1268.2	1.79	1.68–1.90
COPD	857.1	1084.1	1.63	1.46–1.82	1137.6	1279.0	1.58	1.44–1.75
Dementia	848.8	971.9	1.93	1.79–2.09	1424.7	1250.0	1.96	1.86–2.06
Other	888.4	1150.9	1.91	1.80–2.04	1326.2	1405.2	1.91	1.83–2.00
Care Level (p < 0.001)								
Low (ref.)	1022.4	1192.7	1.00		1306.9	1347.6	1.00	
Medium	820.0	1035.2	0.71	0.67–0.75	1189.9	1228.9	0.86	0.83–0.89
High	673.3	1011.3	0.55	0.52–0.58	1309.5	1424.4	0.81	0.78–0.84
Unknown/not specified	297.6	667.7	0.30	0.27–0.33	555.0	1019.3	0.44	0.41–0.48
Multimorbidity (Men: p < 0.001, women: p < 0.05)								
No (ref.)	713.9	1053.0	1.00		1161.6	1334.8	1.00	
Yes	748.3	863.4	1.07	1.02–1.13	1082.4	1055.2	0.98	0.95–1.01
Unknown (no hospital stay)	1145.1	1490.4	0.96	0.89–1.03	1669.4	1636.1	1.03	0.99–1.07
Hospitalisation in the 365 days preceding death (p < 0.001)								
No (ref.)	1131.4	1192.7	1.00		1548.9	1388.5	1.00	
Yes	412.3	757.4	0.36	0.35-0.38	624.1	985.5	0.42	0.41–0.43
Educational level (p < 0.001)								
Low	947.6	1256.8	1.17	1.10–1.23	1308.4	1395.0	1.09	1.06–1.13
Medium (ref.)	700.2	981.5	1.00		1159.8	1269.4	1.00	
High	638.6	836.7	0.98	0.91–1.05	1055.4	1160.6	0.96	0.88–1.05
Unknown	815.3	1023.5	1.10	1.03–1.17	1237.1	1311.3	0.98	0.94–1.02
House or flat owner (p < 0.001)								
Tenant (ref.)	966.7	1263.4	1.00		1439.2	1485.6	1.00	
Owner-occupier	535.3	627.0	0.67	0.64–0.71	823.0	797.2	0.65	0.63–0.67
Marital status (p < 0.001)								
Married (ref.)	648.5	882.1	1.00		1190.7	1435.5	1.00	
Never married	1386.6	1673.6	1.36	1.24–1.48	1531.6	1649.7	1.17	1.10–1.24
Widowed	807.5	1000.5	1.23	1.17–1.29	1219.6	1255.7	1.17	1.13–1.22
Divorced	906.7	1209.4	1.14	1.03–1.25	1175.2	1269.2	0.95	0.89–1.01
Children (p < 0.001)								
Yes (ref.)	680.8	898.2	1.00		1152.6	1236.1	1.00	
No	1133.3	1484.4	1.16	1.08–1.24	1448.4	1552.2	1.10	1.05–1.14
Unknown	1007.4	1211.3	1.16	1.06–1.27	1542.7	1527.8	1.22	1.16–1.28
Language region (Men: p < 0.05; women: p < 0.001)								
German (ref.)	790.9	1090.3	1.00		1264.8	1375.6	1.00	
French	792.4	1016.4	0.95	0.89–1.01	1167.7	1206.5	0.99	0.96–1.03
Italian	763.1	975.0	1.16	1.04–1.31	1405.0	1423.0	1.24	1.16–1.32
Nursing home bed density^a^ (n.s.)			1.00	0.98–1.01			1.01	1.00–1.01

Data source: Swiss Federal Statistical Office, MedStat, SOMED, SNC

IRR = incidence rate ratios, conf interval = 95 % conf. interval, p-values from likelihood ratio tests

^a^average number of nursing home beds per 100 habitants above 65 years (per 106 regions)

Elderly people (at the time of admission) of both sexes stayed significantly shorter in nursing homes compared to younger people. We found significant differences between different causes of death. In comparison to residents with cancer (reference category), residents dying from other chronic diseases like stroke, CHD, COPD or dementia had significantly longer stays. The coefficients of the different care levels suggest for both sexes shorter stays with increasing care level at time of nursing home admission. Men – but not women - with multimorbidity showed slightly but significantly shorter stays. Hospitalisations during the last year of life significantly decreased the length of stay for both sexes. The effects of the educational level are partly significant: We found for example in both sexes significantly longer stays among those with low educational level (compared to medium or high educational level). We found also significant differences according to the marital status. Widowed men and women reported significantly longer stays than married men and women. Having children significantly reduced the length of stays for men and women. The differences between the French and German speaking part of Switzerland were not significant. However, in the Italian speaking part men and women had significantly longer stays than in the German speaking part. But if we look at the average length of stay, men from the German speaking part stayed longer compared to those from the Italian speaking part. We didn’t find a similar effect in women.

## Discussion

As expected, health-related circumstances such as former hospitalizations, specific and multiple chronic diseases or nursing care level had a significant impact on the duration of the last nursing home stay before death. However, the duration of this last nursing home stay was not only dependent on health-related factors alone, but also on a variety of social determinants. Elderly men and women with a higher socio-economic position (SEP, e.g., highly educated, homeowner) had a reduced risk of a long terminal nursing home stay. Especially the effects of homeownership are in line with a recent systematic review and endorse the importance of this indicator [18]. Despite the huge differences in the average length of stay we found very similar gender patterns.

We can interpret these results against the background of studies about morbidity [16] and mortality [11, 13, 28] in old age: people with a high SEP have a health advantage compared to people with low SEP. In addition they presumably have more often the opportunity to stay at home even when health status declines and need of care increases. Homeowners for example can more easily adapt their home in order to remain there even with impaired mobility. Not having a partner (anymore) or grown-up children increases the risk for a longer terminal nursing home stay, probably because a partner and/or own children provide social support and are important informal caregivers.

It has been shown that health-related determinants such as some causes of death (e.g., dementia, stroke) as well as social determinants such as low educational level significantly increase the probability to die in a nursing home [5]. This study provides evidence that the same determinants significantly increase the probability of a longer terminal nursing home stay, too. E. g., dying from dementia – a typical condition of nursing home residents [7, 18, 29, 30] - has a strong effect on both outcomes. However, there are also remarkable differences between the two outcomes. Divorced women, e.g., in our study population did not have significantly longer terminal nursing home stays than married women, irrespective of a compared to married women significantly higher probability to die in a nursing home [5].

In our study, men deceased in a nursing home spent on the average 790 days (median: 382) and women 1250 days (median: 849) in the respective institution before death. This is much longer compared to the average length of nursing home stays in a comparable study from the US. [19], probably pointing to peculiarities of the respective national health systems. A remarkable finding is the influence of the care level at admission time. People with lower care levels at admission had significantly longer stays. There are two possible explanations for this. First, it could be a consequence of better health status at admission: people with a lower care level are generally in better health and have therefore a longer remaining live expectancy. Second, this may concern people with limited resources for providing care at their place of residence, who are therefore more disposed to be early admitted to a nursing home.

Admission to a nursing home is one of life’s major transitions and presumably for a majority of the elderly the last significant decision they make [31]. In contrast, a lot of people dying in institutions have unmet needs (e.g., emotional support, physician communication) about important decisions of end-of-life care [32]. Therefore, the support of elderly people at the admission time of a presumably following nursing home stay should be improved and better evaluated in order to reduce unnecessary and undesired long terminal nursing home stays. Furthermore, an improved support can meet the patient’s and his or her family’s wishes that people can live and die more easily in their preferred place of death.

### Strengths and limitations

One major strength of our study is the national coverage and the extraordinary size of the study population. Another strength is the uniqueness of the data base linking census with mortality and other register data from hospitals and nursing homes. Furthermore, our study gives empirical evidence for several health indicators also considering an extended time period before and therefore only little related with death, which is, to our knowledge a novel approach.

Besides such strengths, the study has some limitations: As usual for secondary data analyses and in particular register data, by far not all desirable information is available and the use of proxy variables with limited validity is inevitable. In this study, we had no data on income or wealth and therefore had to rely on home ownership, an information that has a different meaning depending on place of residence. Also, we had only a limited set of indicators for assessing health status and care level needed. However, the use of such weak measures not necessarily leads to a systematic information bias and rather results in a nondifferential misclassification and therefore in an underestimation of the real association.

## Conclusions

Medical or care needs, but also several not directly health-related factors influence nursing home admission and stays. Social inequalities are not only present with regard to place of death, but also in the duration of the last nursing home stay before death. We found evidence for such inequalities in both sexes. A low educational level, living alone or being tenant as well as a low care level at the beginning of the last nursing home stay increase the risk for longer terminal stays. On the other hand, a high educational level, being homeowner, being married as well as a high care level at the beginning of the last nursing home stay or a hospitalisation in the last year of life decrease the risk for longer stays. Compared to men, the terminal stay of women is on average one third longer, but the role of socio-demographic factors is similar for both sexes. Since nursing homes are often not the preferred place of death and long stays are often expensive, more incentives and support to minimize nursing home admissions for relatively low care-dependent people should become a target of health policy.

## Abbreviations

BIC: Bayesian information criterion
CHD: Coronary heart disease
COPD: Chronic obstructive pulmonary disease
IRR: Incidence rate ratio
ISCED: International standard classification of education
MedStat: Medical statistics of the Swiss hospitals
SEP: Socio-economic position
SNC: Swiss national cohort
SOMED: Statistics of socio-medical institutions
